# Coronavirus 229E with Rhinovirus co-infection causing severe acute respiratory distress syndrome with thrombotic microangiopathy and death during Covid-19 pandemic: lessons to be learnt

**DOI:** 10.4322/acr.2020.194

**Published:** 2020-08-14

**Authors:** Hubert Daisley, Arlene Rampersad, Martina Daisley, Amit Ramdin, Oneka Acco

**Affiliations:** 1 General Hospital San Fernando, Department of Pathology, Trinidad, West Indies; 2 Scarborough General Hospital, Department of Accident and Emergency, Tobago, West Indies

**Keywords:** Coronavirus infections, Rhinovirus, Thrombotic Microangiopathies, Respiratory Distress Syndrome, Adult, SARS virus

## Abstract

We report on a 3-month old infant male who had a seven-days history of fever and rhinorrhea associated with wheezing prior to his death, during the Covid-19 pandemic. Viral testing for Covid-19 (SARS-CoV-2) was negative but was positive for Coronavirus 229E and RP Human Rhinovirus. The pulmonary histological examination showed diffuse alveolar damage along with thrombotic microangiopathy affecting alveolar capillaries. Also, thrombotic microangiopathy was evident in the heart, lungs, brain, kidneys and liver. Thrombotic microangiopathy is a major pathologic finding in Acute Respiratory Distress Syndrome and in the multiorgan failure. This is the first report that illustrates thrombotic microangiopathy occurring in lung, heart, liver, kidney and brain in Acute Respiratory Distress Syndrome with Coronavirus 229E with Rhinovirus co-infection. The clinical presentation and pathological findings in our case share common features with Covid-19.

## INTRODUCTION

Coronavirus 229E with Rhinovirus co-infection, and Covid-19 (SARS-COV-2) each independently can cause severe respiratory tract infection resulting in diffuse alveolar damage and the acute respiratory distress syndrome.^1^ ^-^ ^4^ Coronavirus 229E with Rhinovirus co-infection has hitherto not being reported as causing diffuse alveolar damage and the acute respiratory distress syndrome. Coagulopathies/Immunothrombosis occur in diffuse alveolar damage, the pathologic hallmark of Acute Respiratory Distress Syndrome.^4^ Thrombotic microangiopathy, which was evident in our patient, is the subject of much discussion in Covid-19 disease.^4^ ^-^ ^7^ Coronavirus 229E with Rhinovirus coinfection and Covid-19 share common clinical and pathological findings. Patients suspected of dying from Covid-19 should have confirmatory viral testing.

### Case Report

A 3 months and 26-day old infant male had a seven-day history of fever, rhinorrhea, nasal flaring, and wheezing prior to his death, during the Covid-19 pandemic. No medical attention was sought for the infant’s illness, but he was treated at home with tepid sponge for his fever. There was no complete resolution of his flu-like symptoms as they persisted even onto his death. The infant was delivered at term, vaginally with no adverse events. There was no history of maternal diabetes in pregnancy or HIV. Mother’s antenatal clinic visits were uneventful. The infant was discharged with mother 24 hours after delivery. On the day of the infant’s demise, he was breast fed and placed in his crib on his abdomen. Thirty minutes later the infant was discovered dead by his mother. An autopsy was mandatory.

## AUTOPSY RESULTS

At autopsy, the body was that of a well-nourished 5900 g (50^th^ percentile)^8^ infant male with head circumference 38 cm (<5^th^percentile).^8^ There was central and peripheral cyanosis. There were no external congenital defects. The thymus weighed 52 g (10±5 g)^8^ and was grossly unremarkable. There was no hyperemia of the trachea or milk curd particles within. There were serous pleural effusions, of 40 ml and 30 ml respectively in the right and left pleural cavities. The right lung weighed 98 g and was consolidated. The left lung weighed 68 g and all lobes floated in water (combined weight 89±23 g).^8^

**Figure 1:**
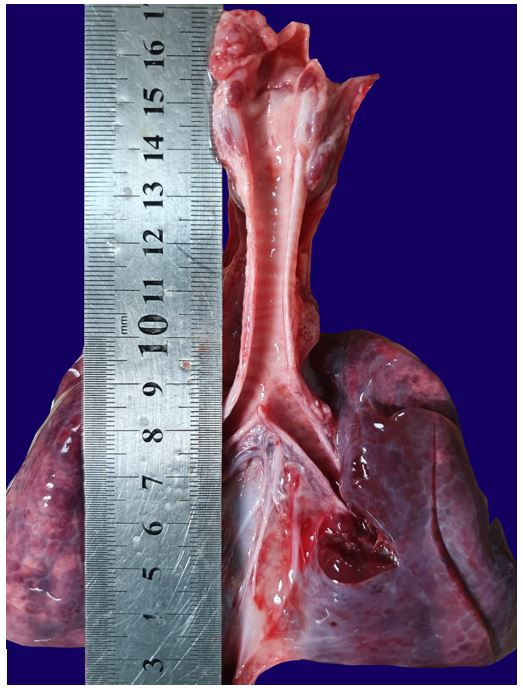
Gross view of the larynx, trachea, left and right lungs, the latter with consolidation. Note the absence of signs of aspiration.

The heart weighed 46 g (30±7)^8^ and there were no congenital cardiac defects. (Figure 1) The liver weighed 460 g (179±41 g),^8^ spleen 22 g (16±5 g),^8^ and each kidney 26 g (combined weight 45±10)^8^ were congested. White curds were seen in the stomach but there was no abnormality in the gastrointestinal tract.

**Figure 2:**
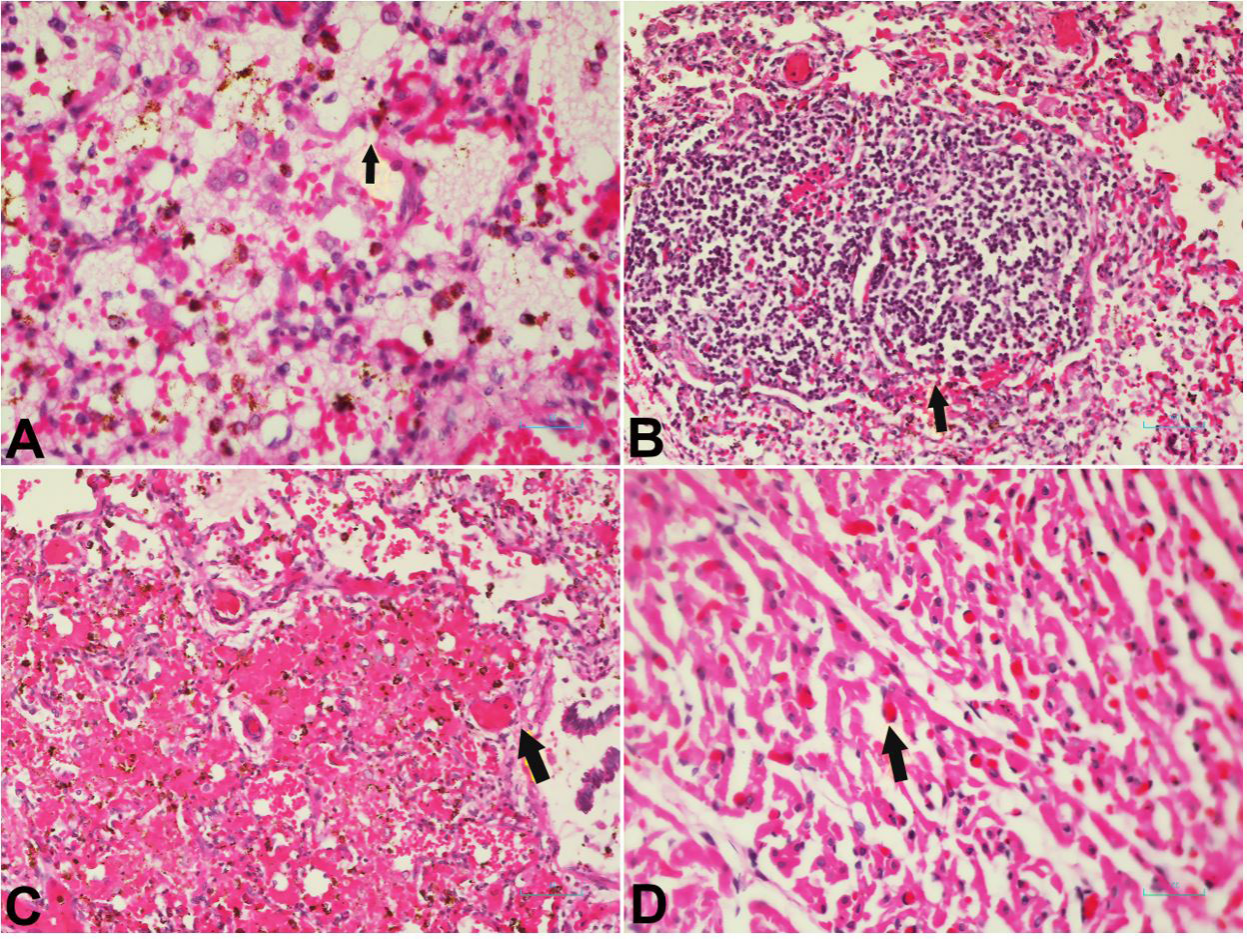
Photomicrographs of the lung and myocardium. **A –** Lung showing diffuse alveolar damage with denudation of epithelial cells, and hyaline membrane formation (arrow); **B –** Lung with focal areas with moderate infiltrate of lymphocytes within the alveoli (arrow); **C –** Thrombotic microangiopathy present in alveolar capillaries (arrow) and smaller pulmonary vessels; **D –** Thrombotic microangiopathy present in the myocardium. Here arrow points to microthrombi within a small vessel of the myocardium.

**Figure 3:**
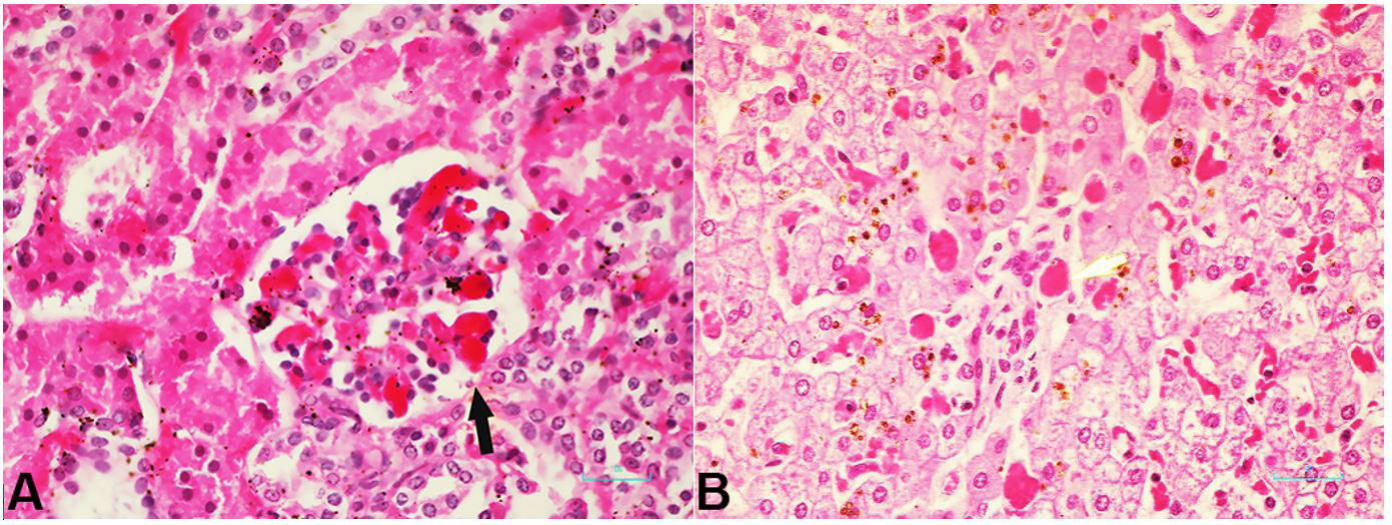
Photomicrographs of the kidneys and liver. **A –** Kidney showing microthrombi within glomerular capillaries (arrow) (H&E X 400); **B –** Microthrombi within portal vessels (arrow) and sinusoids of the liver.

The brain, 288 g (567±81 g),^8^ was edematous. In view of the current Covid-19 pandemic, swabs from the right lung were sent for virological and bacteriological studies. Sections from all tissues examined were processed for histological examination using H&E stain. Fifteen viruses were tested for, using PCR. SARS-CoV-2 was negative. Coronavirus 229E (CoV-229E) was positive and also Human Rhinovirus. There was no bacterial growth from the consolidated right lung. The lung showed diffuse alveolar damage with denudation of epithelial cells, hyaline membrane formation (Figure 2A) and focal areas with moderate infiltrate of lymphocytes within alveoli (Figure 2B). Thrombotic microangiopathy was seen in alveolar capillaries and smaller pulmonary vessels (Figure 2C). The alveolar spaces were filled with edema, macrophages, giant cells and diffuse hemorrhage in focal areas. There was no neutrophilic infiltration within the alveolar spaces or walls as would be expected in secondary bacterial infection. Thrombotic microangiopathy was seen in the myocardium (Figure 2D), kidneys in glomerular tufts and interlobular arteries (Figure 3A), liver in portal tracts and sinusoids (Figure 3B) and brain.

## DISCUSSION

We present, herein, a fatal case of viral pneumonia caused by CoV-229E with Rhinovirus co-infection for which there was no medical intervention. Hence the pathologic findings of this case can only be attributed to the CoV-229E and Rhinovirus infections. Asphyxia from mucus blocking the upper airways might have contributed to the infant’s demise.

This infant had a viral pneumonia with CoV-229E and Rhinovirus co-infection as the causative agents. Acute lower respiratory infections, such as pneumonia and bronchiolitis are the leading cause of morbidity and mortality in children under the age of 5 years.^9^ Both CoV-229E and Rhinovirus independently can cause pneumonias and Acute Respiratory Distress Syndrome (ARDS) in immunocompromised and healthy hosts.^10^ ^-^ ^12^ Of more concern is the fact that human Coronavirus with Rhinovirus coinfection is a risk factor for severe respiratory disease and admission to Pediatric intensive care unit.^13^ An increase level of multiple respiratory viral pathogens coinfection has been isolated in several studies of viral pneumonia. These coinfections are partly responsible for the severity of acute viral respiratory disease in children.^14^ ^-^ ^16^

Another risk factor associated with mortality in infants with lower respiratory infections is hypoxemia.^17^ The infant, in discussion, was cyanotic and must have been hypoxic at the latter stage of his illness. This infant, although severely ill, was not taken for medical care, at a hospital or medical clinic probably because of the Government's “stay at home” guidance instituted during the Covid-19 pandemic. Maternal education and socioeconomic status might also have contributed to the infant’s demise.^18^

CoV-229E with Rhinovirus coinfection produced diffuse alveolar damage (DAD), and acute respiratory distress syndrome, in our case. In COVID-19 deaths reported in the current pandemic, DAD is the pathological hallmark of ARDS.^3^ ^,^ ^5^ Hence there are no distinguishing features either clinically or pathologically between Covid- 19 and CoV-229E with Rhinovirus coinfection pneumonia. Viral testing is the key point to unveil the etiology.

It would be interesting to learn if viral coinfection(s) play a role in the severity of Covid-19 in the present pandemic.

The human alveolus is made up of a respiratory bronchiole, alveolar macrophages, type I and type II pneumocytes resting on a basement membrane, which is shared with alveolar capillary endothelial cells. It only calls to reason that injury to alveoli by whatsoever agent would generate similar responses viz, destruction of epithelial and endothelial cells, the production of hyaline membrane and fibrin thrombi, epithelial cells hyperplasia, hemorrhage, edema, and the migration of macrophages and giant cells within the alveoli. The extent of the response to the damaged alveolus would depend upon the host immunity and the persistence of the injurious agent at the alveolar site. An exaggerated innate host immune response to epithelial cells injury might invoke the cytokine storm, which might be more injurious than protective to the alveoli, and ARDS and multiorgan failure might occur.^19^ ^-^ ^21^ Hence SARS-COV-2 virus and CoV-229E with Rhinovirus co-infection, produce similar damage of alveoli and induce an identical innate immunological response. Thrombotic microangiopathy viz fibrin thrombi, a feature of diffuse alveolar damage, was a major pathological finding in our case, for this was evident within the alveoli capillaries, (Figure 2C), alveolar wall, and small vessels in the lung.^5^ ^,^ ^22^ The myocardium, the glomerular tufts and interlobular arteries in the kidney, liver and in the brain. This immunothrombotic process is an innate immunological response that occurs following alveolar capillary endothelial cell injury. Hyaline membrane, which consists of fibrin deposits along the damaged alveolar wall, represents similar innate immune thrombotic response following alveolar wall injury.^4^ ^-^ ^6^ ^,^ ^23^ ^-^ ^25^ The release of tissue factor into the systemic circulation during endothelial cell and alveolar wall injuries, initiates the thrombotic microangiopathy in multiple organs, which contributes to the multiorgan failure often seen in acute respiratory distress syndrome. Symmers^26^ in 1952, in his review of thrombotic microangiopathy, reported on two of his cases, the first of which started off as a febrile cold, and the second post inguinal herniorrhaphy. Both cases showed disseminated thrombotic microangiopathy in multiple organs. All cases reported in his review were fatal. Thrombotic microangiopathy is life threatening, resulting in ischemic multiorgan failure as seen in our case, and characterized by high mortality rates despite the appropriate treatment.^27^ The case in discussion is the first report, which illustrates immunothrombosis/thrombotic microangiopathy occurring in multiple organs in the acute respiratory distress syndrome and viral pneumonia caused by CoV-229E with Rhinovirus coinfection.

Thrombotic microangiopathy affecting alveolar capillaries was a major catastrophic event in our case (Figure 2C). This phenomenon on its own can lead to severe respiratory distress and respiratory failure. Thrombotic microangiopathy also lends a plausible explanation for the severity of the Acute Respiratory Distress Syndrome in viral pneumonias.

The infant, under discussion, was severely ill based upon the pathological findings. Multiple factors contributed to his cause of death namely, severe viral pneumonia with CoV-229E and Rhinovirus co-infection, diffuse alveolar damage, acute respiratory distress syndrome with respiratory failure and multiple organ failure from thrombotic microangiopathy that were demonstrated in the lungs, kidneys, liver, myocardium and brain, and confluent alveoli thrombosis.

CoV-229E with Rhinovirus coinfection and Covid-19 (SARS-CoV-2), cause diffuse alveolar damage and severe acute respiratory distress syndrome as the underlying pulmonary event. Thrombotic microangiopathy is one of the findings in DAD.^5^ It has been shown that therapy aimed at the immunothrombosis has an ameliorating effect in ARDS in patients in the Covid-19 pandemic.^4.6,7^

The Coronavirus, CoV-229E belongs to the same family of Coronaviridae as SARS-CoV-2, the virus that is presently causing the COVID 19 pandemic. SARS-CoV-2 and CoV-229E might have shared antigenic structures. Neutralizing antibodies to CoV-229E infection might produce some protection against the SARS-CoV-2 infection.^28^ ^-^ ^30^

CoV-229E is a frequent cause of upper respiratory tract infection in infants. This might be the reason why children are resistant to contracting Covid-19, for their neutralizing antibodies to CoV-229E infection might be offering them some protection from SARS-CoV-2. It might also offer an explanation to the subclinical manifestation of Covid19 in a significant proportion of the worlds’ population, for there might be shared neutralizing antibodies to SARS-CoV-2 from previous CoV-229E and other coronavirus infection in this subclinical population. It is also interesting to note that both Coronavirus 229E and SARS-CoV-2 are implicated in Kawasaki disease.^31^ ^,^ ^32^

## CONCLUSION

Coronavirus 229E with Rhinovirus coinfection presents with similar clinical and pathological manifestations as Covid-19 and cause diffuse alveolar damage, the pathological hallmark of Acute Respiratory Distress Syndrome. In the present Covid-19 pandemic, virological studies should be done on all deaths suspected of Covid-19 disease to elucidate the true nature of the infectious agent, for not all respiratory deaths in the Covid-19 pandemic are attributed to Covid-19 as the case in discussion demonstrates. It would be interesting to see whether viral coinfections in the Covid-19 pandemic influence the severity of the disease.

Immunothrombosis, caused by Coronavirus 229E with Rhinovirus coinfection and Covid-19 disease, may become exaggerated during cytokine storm and thereby cause fibrin deposits within alveolar wall and thrombotic microangiopathy in the lung, myocardium, kidney, liver, brain and other organs, which subsequently cause multiorgan failure.

This is the first report illustrating thrombotic microangiopathy of the lungs, kidneys, myocardium, liver and brain occurring in a Coronavirus 229E with Rhinovirus coinfection pneumonia. Therapy aimed at thrombotic microangiopathy might be ameliorative in the acute respiratory distress syndrome occurring in viral pneumonia.^4^ ^,^ ^6^ ^,^ ^7^

## References

[1] Ngu S, Pervaiz S, Avula A, Chalhoub M (2019). Rhinovirus-induced rapidly progressing acute respiratory distress syndrome in an immunocompetent host. Cureus.

[2] Vassilara F, Spyridaki A, Pothitos G, Deliveliotou A, Papadopoulos A (2018). A rare case of human Coronavirus 229E associated with acute respiratory distress syndrome in a healthy adult. Case Rep Infect Dis.

[3] Xu Z, Shi L, Wang Y (2020). Pathological findings of COVID-19 associated with acute respiratory distress syndrome. Lancet Respir Med.

[4] Whyte CS, Morrow GB, Mitchell JL, Chowdary P, Mutch N (2020). Fibrinolytic abnormalities in acute respiratory distress syndrome (ARDS) and versality of thrombolytic drugs to treat COVID-19. J Thromb Haemost.

[5] Dolhikoff M, Durate-Neto AN, Monteiro RA (2020). Pathological evidence of pulmonary thrombotic phenomena in severe Covid-19. J Thromb Haemost.

[6] Connors JM, Levy JH (2020). Levy thromboinflammation and the hypercoagulability of COVID‐19. J Thromb Haemost.

[7] Wang J, Hajizadeh N, Moore EE (2020). Tissue Plasminogen Activator (tPA) treatment for COVID‐19 Associated Acute Respiratory Distress Syndrome (ARDS): a case series. J Thromb Haemost.

[8] Connolly AJ, Finkbeiner WE, Ursell PC, Davis RL (2016). Autopsy pathology: a manual and atlas.

[9] Nair H, Simões EA, Rudan I (2013). Global and regional burden of hospital admissions for severe acute lower respiratory infections in young children in 2010: a systematic analysis. Lancet.

[10] Hai T, Bich V, Ngai L (2012). Fatal respiratory infections associated with rhinovirus Outbreak, Vietnam. Emerg Infect Dis.

[11] Imakita M, Shiraki K, Yutani C, Ishibashi-Ueda H (2000). Pneumonia caused by Rhinovirus. Clin Infect Dis.

[12] Kennedy JL, Turner RB, Braciale T, Heymann PW, Borish L (2012). Pathogenesis of rhinovirus infection. Curr Opin Virol.

[13] Matsuno AK, Gagliardi TB, Paula FE (2019). Human coronavirus alone or in co-infection with rhinovirus C is a risk factor for severe respiratory disease and admission to the paediatric intensive care unit: a one-year study in Southeast Brazil. PLoS One.

[14] Calvo C, García-García ML, Blanco C (2008). Multiple simultaneous viral infections in infants with acute respiratory tract infections in Spain. J Clin Virol.

[15] Peng D, Zhao D, Liu J (2009). Multipathogen infections in hospitalized children with acute respiratory infections. Virol J.

[16] Upadhyay BP, Banjara MR, Shrestha RK, Tashiro M, Ghimire P (2018). Etiology of coinfections in children with Influenza during 2015/16 winter season in Nepal. Int J Microbiol.

[17] Lazzerini M, Sonego M, Pellegrin MC (2015). Hypoxaemia as a mortality risk factor in acute lower respiratory infections in children in low and middle-income Countries: systematic review and meta-analysis. PLoS One.

[18] Sonego M, Pellegrin MC, Becker G, Lazzerini M (2015). Risk factors for mortality from acute lower respiratory infections (ALRI) in children under five years of age in low and middle-income countries: a systematic review and meta-analysis of observational studies. PLoS One.

[19] Gralinski LE, Baric RS (2015). Molecular pathology of emerging coronavirus infections. J Pathol.

[20] Nicholls JM, Poon LL, Lee KC (2003). Lung pathology of fatal severe acute respiratory syndrome. Lancet.

[21] Beasley MB (2010). The pathologist’s approach to acute lung injury. Arch Pathol Lab Med.

[22] Ackermann M, Verleden SE, Kuehnel M (2020). Pulmonary vascular endothelialitis, thrombosis, and angiogenesis in Covid-19. N Engl J Med.

[23] Frantzeskaki F, Armaganidis A, Orfanos SE (2017). Immunothrombosis in acute respiratory distress syndrome: cross talks between inflammation and coagulation. Respiration.

[24] Greene R, Lind S, Jantsch H (1987). Pulmonary vascular obstruction in severe ARDS: angiographic alterations after i.e. fibrinolytic therapy. AJR Am J Roentgenol.

[25] Hill NS, Roberts K, Preston I (2010). Pulmonary vasculopathy in acute respiratory distress syndrome: something new, something old…. Am J Respir Crit Care Med.

[26] Symmers W (1952). Thrombotic microangiopathic haemolytic anaemia. BMJ.

[27] Joly BS, Zheng XL, Veyradier A (2018). Understanding thrombotic microangiopathies in children. Intensive Care Med.

[28] Marra MA, Jones SJ, Astell CR (2003). The Genome sequence of the SARS-associated coronavirus. Science.

[29] Reed SE (1984). The behaviour of recent isolates of human respiratory coronavirus in vitro and in volunteers: evidence of heterogeneity among 229E‐related strains. J Med Virol.

[30] He Y, Zhou Y, Wu H, Kou Z, Liu S, Jiang S (2004). Mapping of antigenic sites on the nucleocapsid protein of the severe acute respiratory syndrome coronavirus. J Clin Microbiol.

[31] Shirato K, Imada Y, Kawase M, Nakagaki K, Matsuyama S, Taguchi F (2014). Possible involvement of infection with human coronavirus 229E, but not NL63, in Kawasaki disease. J Med Virol.

[32] Verdoni L, Mazza A, Gervasoni A (2020). An outbreak of severe Kawasaki-like disease at the Italian epicentre of the SARS-CoV-2 epidemic: an observational cohort study. Lancet.

